# Short-Term Changes in Nutrition Literacy, Dietary Engagement, and Reported Food Habits Among Adolescents After One Session with a Gamified Digital Nutrition-Education Platform: A Single-Arm Pre–Post Study

**DOI:** 10.3390/nu18203447

**Published:** 2026-10-20

**Authors:** Anca Georgiana Ispas, Alexandra Lacurezeanu, Alina-Ioana Forray, Andreea Rusu, Dumitru Petreuș, Răzvan Mircea Cherecheș

**Affiliations:** 1 Department of Public Health, College of Political, Administrative and Communication Sciences, Babes-Bolyai University, 400376 Cluj-Napoca, Romania; georgiana.ispas@publichealth.ro (A.G.I.);; 2 Faculty of Food Science and Technology, University of Agricultural Sciences and Veterinary Medicine, 400372 Cluj-Napoca, Romania; 3 Discipline of Public Health and Management, Department of Community Medicine, “Iuliu Hațieganu” University of Medicine and Pharmacy, 400012 Cluj-Napoca, Romania; 4 Astralis Solutions S.R.L., Strada Colinei 20, 400520 Cluj-Napoca, Romania; 5 Asociația Wello, 400686 Cluj-Napoca, Romania

**Keywords:** gamification, digital health intervention, nutrition literacy, dietary behaviour, food habits, adolescents, school-based nutrition education, Romania

## Abstract

**Background/Objectives**: No study has evaluated a gamified nutrition-education platform for adolescents in Eastern Europe. This pilot study described short-term changes in nutrition literacy, dietary engagement and reported food habits after one session with AHOPE, an automated gamified platform, among Romanian adolescents. **Methods**: In a single-arm pre–post study, 332 adolescents aged 10–19 years began one session and 155 (46.7%) completed it, answering three scales, including an adapted forced-choice Adolescent Food Habits Checklist, immediately before and after. Paired tests used Benjamini–Hochberg correction; moderation and predictor analyses were exploratory. **Results**: All six outcome scores were higher after the session (all p_BH < 0.001; *dz* 0.42–0.73, largest for nutrition literacy). Of 23 food-habit items, 17 shifted toward the healthier response, with the largest shifts for the foods taught in the modules. Interaction tests gave no evidence that change differed by sex, age band or family affluence (all p_BH ≥ 0.90), although power was limited (low-affluence group, *n* = 18). **Conclusions**: Among completers, who were more affluent than non-completers, self-reported nutrition literacy, dietary engagement and food-habit responses were higher immediately after one session. These changes may reflect short-term intentions; the design cannot establish causation, persistence or behavioural change, which require a controlled trial with delayed follow-up.

## 1. Introduction

Between 1990 and 2022, the age-standardised prevalence of obesity among children and adolescents aged 5 to 19 years rose from 1.7% to 6.9% in girls and from 2.1% to 9.3% in boys, reaching approximately 160 million individuals [1], within a wider group of more than 390 million living with overweight in 2022 [2]. Adolescence (10 to 19 years) is a decisive stage in this trajectory, since dietary patterns established during this period moderately persist into adulthood [3,4]. At the same time, adolescents gain autonomy and agency over what they eat, and their food choices become increasingly shaped by the food environment and peers rather than by parents [5], making the delivery format of a nutrition intervention as important as its content.

In Europe, adolescent diet remains far from guideline recommendations. In 2021/22, only 38% of adolescents consumed the recommended daily servings of fruit and vegetables, with even lower rates among boys (35%); more than half of 15-year-old participants (56% of boys and 51% of girls) reported not eating fruit or vegetables daily, and adolescents from less affluent families consistently ate less of both (34% vs. 47% for girls, 30% vs. 42% for boys) [6]. The transition toward healthier dietary patterns has also been uneven: it was already under way in many Western European countries in 1990–2000, whereas in Eastern Europe, it began later and less consistently, with an increase in fruit and vegetable intake reported in 9 of 16 Western countries but only 2 of 7 Eastern European countries during the 2000s [7]. In Romanian children aged 5 to 17 years, 25.1% have excess weight [8], and among older pupils, vegetable consumption is lower and chaotic eating patterns are more common [9]; the pattern extends into young adulthood (18 to 30 years), where compulsive eating and soft-drink intake are the correlates most consistently associated with higher BMI [10]. The same population is intensely digital: among 900 Transylvanian adolescents, 98.7% owned a mobile phone and 65.8% exceeded two hours of daily recreational screen exposure [11], and across 41 countries, problematic social media use was associated with substantially lower odds of good dietary intake in both sexes [12], making the digital environment at once a risk factor for the adolescent diet and an underused channel for reaching it.

Nutrition education is the most common response to this gap between adolescent diets and dietary guidelines. However, knowledge alone is a weak lever: in a systematic review of 13 studies in children and adolescents, the association between nutrition knowledge and dietary behaviour was inconsistent, and 4 studies found no significant relationship [13]. Current interventions therefore target nutrition literacy, the capacity to obtain, process and critically evaluate nutrition information [14], together with engagement in dietary behaviour and everyday food habits; in adolescents, higher nutrition literacy directly predicts healthier food habits measured with the same checklist used in the present study [15]. Classroom-based programmes can produce measurable short-term gains. In a quasi-experimental pilot with 127 fifth-grade pupils in two Romanian municipalities, a teacher-delivered curriculum significantly improved the reading and understanding of food labels, the comprehension of dietary recommendations, resistance to unhealthy food choices, and willingness to try new vegetables [16]. However, this format depends on a trained instructor and a fixed lesson period, while the size of the improvement across such programmes is unrelated to the number of tools, lesson duration or programme length [17], which suggests that a lighter, automated format need not deliver less. These observations have shifted attention toward automated digital delivery.

Gamification, defined as the design of systems that afford experiences and motivations similar to those of games to influence behaviour [18], offers a delivery format aligned with how adolescents already engage with digital media. Its theoretical basis lies in self-determination theory, in which motivation is sustained when the needs for autonomy, competence and relatedness are supported [19]. For nutrition, games and gamified applications improve knowledge and self-reported eating behaviour [20], and a meta-analysis of gamified interventions found a large improvement in nutrition-knowledge scores in favour of gamified conditions [21], whereas a meta-analysis of 20 serious-game trials found a moderate effect on physical activity but no significant effect on dietary change [22]. In the most recent synthesis of 34 randomised trials of mobile and web-based interventions in this age group, most of them game-based, nutrition knowledge improved in two-thirds of studies and fruit intake in half, while anthropometric outcomes remained unchanged [23]. Uncontrolled pre–post designs remain common at this stage of the field, as in a gamified school programme in Milan, where the dietary score of 184 adolescents improved from 7.6 to 8.2 over one academic year [24].

However, the literature is geographically concentrated. Of the 20 trials pooled in the serious-games meta-analysis, half were conducted in the United States, and the remainder in eight other Western countries [22]; the gamification meta-analysis likewise drew mostly on studies from the United States and Italy [21]. To our knowledge, no study has evaluated a gamified nutrition-education platform among adolescents in Romania or elsewhere in Eastern Europe. Gamified digital education has been evaluated in Romanian schools in other health domains, including a cross-national digital-game-based health-education platform [25] and a Kahoot-supported physical-literacy intervention [26], but neither targets nutrition, and neither uses a fully automated curriculum without a teacher in the room. AHOPE (Adolescents’ Healthy Options and Positive Eating) was developed to address this gap by converting the previously piloted, teacher-delivered curriculum [16] into a fully automated, gamified digital platform. This pilot pre–post evaluation describes the change in nutrition literacy, dietary engagement and reported food choices from immediately before to immediately after a single AHOPE session, and explores whether this change differed by sex, age and family affluence. A single-arm design of this kind can show whether the automated curriculum can be delivered and completed and estimate the size of short-term change as groundwork for a controlled trial; it cannot test effectiveness.

## 2. Materials and Methods

### 2.1. Study Design

A single-arm, pre–post study was conducted to evaluate short-term changes in nutrition literacy, engagement in dietary behaviour and food-habit choices after one session with the AHOPE gamified digital platform. Each participant completed the same self-administered questionnaire twice, immediately before and immediately after using the application. The platform enforces this sequence technically, since the questionnaire and the modules share the same login, and the post-questionnaire link does not become active until all four end-of-module quizzes are recorded as completed. Pupils accessed the platform on their personal smartphones in the classroom, under teacher supervision during the school term or outside school during the summer holiday. The study was carried out within the AHOPE project, implemented by Babeș-Bolyai University Cluj-Napoca in partnership with Astralis Solutions S.R.L. Data were collected between 31 March and 19 August 2026 after ethics approval was obtained on 25 March 2026. The study is reported in accordance with the TREND statement for non-randomised evaluations; the completed checklist is provided as Table S1.

### 2.2. Participants and Recruitment

Participants were pupils aged 10 to 19 years enrolled in Romanian schools. Recruitment started in partner schools in Cluj-Napoca with an established collaboration agreement with Babeș-Bolyai University and was extended nationally through the research team’s network of teachers and through online sharing of the platform link in parents’ groups, applying the same consent procedure approved by the Research Ethics Subcommittee (Section 2.3). Eligible pupils had access to a digital device; pupils were excluded if they or their parent or legal guardian declined consent, or if a language barrier or a severe cognitive disability prevented them from understanding the questionnaire. Age was based on self-report in years; for records with an internally inconsistent or unparseable date of birth, the self-reported age was used wherever age was required.

### 2.3. Ethical Approval

The study was conducted in accordance with the Declaration of Helsinki and the Oviedo Convention on Human Rights and Biomedicine. The protocol was reviewed and approved by the Research Ethics Subcommittee of Babeș-Bolyai University Cluj-Napoca (approval No. 4299/25.03.2026; request No. 143/11.03.2026). Data were collected through the AHOPE platform, pseudonymised through a unique participant code assigned within the platform, and processed in accordance with Regulation (EU) 2016/679 (GDPR) and Romanian Law No. 190/2018; the pseudonymised research dataset was accessed for analysis only by the university research team.

The research team first obtained institutional agreement from each school. Consent was then obtained at three levels, following the procedure approved by the Research Ethics Subcommittee. For participants under 18 years, parents or legal guardians received information sheets and consent forms through the schools’ online channels and returned a digital consent form covering the processing of the child’s data; adolescents then gave assent within the platform before accessing any content. Participants aged 18 years or older gave their own informed consent within the platform, including explicit consent to data processing under Articles 6(1)(a) and 9(2)(a) of Regulation (EU) 2016/679. Participation was voluntary, participants could skip any item or withdraw at any point, and no compensation was offered.

### 2.4. The AHOPE Intervention

The AHOPE session consisted exclusively of a digital platform, described by the project team as a “digital companion”, which delivers a nutrition-education curriculum through interactive quizzes, missions, rewards, and competitive and collaborative features within an interface designed for children and adolescents. The curriculum is the one previously validated in a teacher-delivered pilot in Romanian schools [16]; AHOPE retains this content but replaces classroom instruction with a fully automated gamified format. No teacher-led lesson, workshop or other educational activity accompanied the platform; the classroom served only as a supervised setting in which pupils accessed the application on their personal smartphones. A session comprised four modules (“Macro- and Micronutrients,” “Water, Oxygen and Sleep,” “Ultra-Processed Foods and Food Labels,” and “Kitchen Basics”), each 7 to 8 min long and ending with a retakeable 10-item quiz giving instant feedback and a bronze, silver or gold badge (6 to 7, 8, or 9 to 10 of 10 points); module scores summed to an overall badge (maximum 40 points). Detailed descriptions of the module content, the quiz items and the badge system are provided in the Supplementary Materials (Tables S2–S5). The “competitive and collaborative features” were this badge and scoring system; the platform had no peer leaderboards or team-based tasks. No quiz item reproduced the wording of an S-NutLit, EDB or AFHC item. The curriculum and the outcome measures nevertheless cover overlapping content domains, in particular ultra-processed foods, sugar-sweetened drinks, fried food and fruit and vegetable intake, so part of the post-session change in these domains may reflect exposure to the taught material rather than a change in habit.

### 2.5. Measures

The AHOPE questionnaire was the only data-collection instrument. It was self-administered within the digital platform, took approximately 20 min per administration, and combined a sociodemographic, anthropometric and lifestyle module with three validated scales. The EDB was developed and validated in young adolescents [27], and the AFHC, originally validated in adolescents aged 13 to 16 years [28], has been applied in school-based samples spanning ages 9 to 19 [29,30]. The S-NutLit was developed for young adults [14]; the internal consistency of all three scales in the present sample was high at both administrations (Section 3.3). The Romanian versions were produced by two bilingual researchers from the author team (A.-I.F. and A.R.) following the WHO process for translating and adapting instruments, with forward translation followed by consensus back-translation [31]. The Romanian versions were then checked against the original English instruments item by item, across all form elements, covering item wording, response options, scale anchors, grid structures, and skip logic; no discrepancy affecting item meaning was identified, and minor wording differences were harmonised. No cognitive interviews or pilot testing of the Romanian versions were conducted, and their factor structure was not examined in this sample (Section 4.5).

#### 2.5.1. Sociodemographic, Anthropometric and Lifestyle Module

Participants reported their date of birth, height and weight, from which body mass index (BMI, kg/m^2^) was calculated. Sex (male/female) was recorded by the AHOPE platform and was used in all analyses, reported as boys and girls. Additional items covered current weight-management intention (trying to lose, gain or maintain weight, or doing nothing), the number of days per week with at least 60 min of moderate-to-vigorous physical activity, daily recreational screen time, and prior participation in nutrition-education activities. Socioeconomic status was assessed with the six-item Family Affluence Scale III (FAS III) [32], summed to a 0 to 13 total and categorised as low (0 to 6), medium (7 to 9) or high (10 to 13) affluence.

#### 2.5.2. Nutrition Literacy (S-NutLit)

Nutrition literacy was measured with the Short Nutrition Literacy Scale (S-NutLit) [14], comprising 11 items rated on a 5-point scale and covering information-related and expert-related nutrition skills.

#### 2.5.3. Engagement in Dietary Behaviour

Engagement in dietary behaviour was assessed with the six-item Engagement in Dietary Behaviour (EDB) scale [27], rated from 1 (“strongly disagree”) to 6 (“strongly agree”). Two items each address engagement at the personal, social and global level, yielding a total score (6–36) and three two-item facet scores.

#### 2.5.4. Food Habits (Adapted AFHC)

Food habits were assessed with an adapted version of the 23-item Adolescent Food Habits Checklist (AFHC) [28], a true/false inventory of everyday food choices such as avoiding fried food, choosing low-fat snacks and eating fruit daily. In the original instrument, 10 items (1, 6, 7, 11, 16, 17, 18, 19, 20 and 21) offer a third response for respondents to whom the item does not apply (for example, “I never have lunch away from home”); such responses are excluded and the total is prorated to 23 items [28]. The AHOPE platform presented every item as a forced true/false choice without this option, so all participants answered all 23 items and no proration was applied. This adapted format has not been validated against dietary intake, and its scores are not directly comparable with those obtained with the original checklist. Five items worded in the unhealthy direction (3, 8, 14, 18 and 21) were reverse-scored before summing, giving a total of 0–23, with higher scores reflecting healthier reported choices.

### 2.6. Statistical Analysis

The data were analysed as paired observations. Internal consistency of each scale was estimated at both administrations with Cronbach’s α and McDonald’s ω. Baseline characteristics were compared between boys and girls with Welch’s *t*-test for continuous variables and the chi-squared test for categorical variables. Participants who completed the post-questionnaire and those who did not were compared on all pre-questionnaire variables with the same tests. BMI was converted to age- and sex-specific z-scores using the WHO 2007 growth reference for ages 5 to 19 years [33]. The normality of within-person difference scores was examined with the Shapiro–Wilk test. Pre–post change in each of the six outcome scores (S-NutLit, EDB total, EDB personal, EDB social, EDB global, AFHC total) was tested with paired-samples *t*-tests and confirmed with the Wilcoxon signed-rank test. Effect sizes were expressed as Cohen’s *dz* with 95% confidence intervals and as the matched-pairs rank-biserial correlation. Item-level change on the AFHC was tested with McNemar’s test for paired binary data. The association between total module quiz score and the change in each outcome was assessed with Pearson correlations. The Benjamini–Hochberg false-discovery-rate correction was applied within three families: across the six outcomes for the main pre–post tests, across the 23 AFHC items for the item-level tests, and across the 18 time × moderator interaction tests (six outcomes by three moderators); adjusted *p*-values are reported as p_BH.

The primary analysis was the pre–post comparison of the six outcome scores, with Benjamini–Hochberg correction across the six outcomes. The AFHC item-level McNemar tests were secondary. Moderation by sex, age band and FAS III category, the ANCOVA-style predictor models and the correlations between quiz score and change were exploratory: subgroup and regression analyses were listed in the approved analysis plan, but the specific models were chosen at the analysis stage, the study was not registered and the sample was not sized for subgroup comparisons. A non-significant interaction is not taken as evidence that change was equal across subgroups. Moderation of the pre–post change by sex, age band (10 to 14 vs. 15 to 19 years) and FAS category was tested with linear mixed-effects models (random intercept per participant, time × moderator interaction, Satterthwaite degrees of freedom), with simple contrasts from estimated marginal means. Predictors of the post-intervention score were examined with ANCOVA-style regression on baseline score, sex, age, FAS III score, physical-activity days and prior nutrition-education exposure, checking multicollinearity with variance inflation factors. No a priori sample-size calculation was performed; recruitment continued for the duration of the data-collection window and the achieved sample determined the precision. A sensitivity analysis indicates that with *n* = 155, α = 0.05 (two-sided) and 80% power, the smallest detectable within-person effect is *dz* = 0.23. Observed power was not computed. Statistical significance was set at α = 0.05 (two-sided). Analyses were performed in R version 4.6.1 [34] using psych, lme4/lmerTest, emmeans, effectsize, rstatix, broom/broom.mixed, car and ggplot2.

### 2.7. Use of Generative Artificial Intelligence

During the preparation of this manuscript, the authors used Anthropic’s Claude (Claude Sonnet 5 and Claude Fable 5.1) as a writing and analytical aid: language polishing (fixing grammar, improving clarity and flow), outlining (structuring preliminary ideas and organising section headings), verification support for citations and reference details, and data-coding assistance in R (assisting with coding scripts and syntax during data preparation, fully documented). AI served as an aid only; the study design, data collection, statistical analysis, interpretation, and all intellectual contributions remained with the human authors. The authors reviewed and edited all AI-assisted outputs and take full responsibility for the content of this publication.

## 3. Results

### 3.1. Participant Characteristics

The analytic sample came from 74 schools in 19 cities; 81 participants (52.3%) attended 21 schools in Cluj-Napoca, and 74 participants attended 53 schools in 18 other cities across Romania.

**Figure 1:**
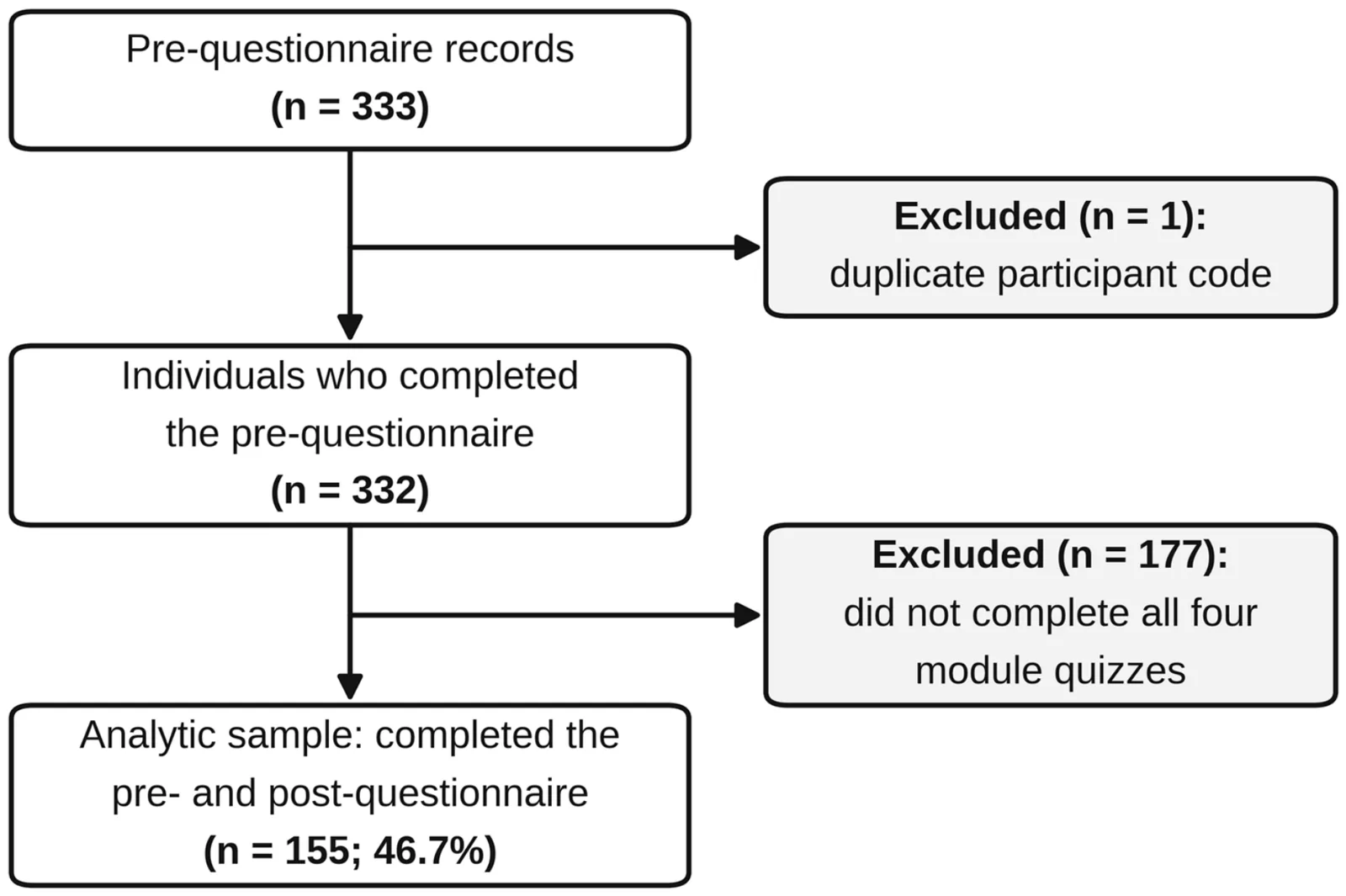
Participant flow from pre-questionnaire records to the analytic sample.

Data collection produced 333 pre-questionnaire records; 1 participant code appeared twice, the later record was discarded and the earlier retained, giving 332 individuals (Figure 1). Of these, 155 (46.7%) completed the post-questionnaire and form the analytic sample. Because the post-questionnaire opened only after all four module quizzes were completed, non-completion means that the session was not finished, and the analytic sample is a selected subgroup of those who started. Completers (*n* = 155) and non-completers (*n* = 177) were compared on the pre-questionnaire variables (Table S10). They did not differ in age (15.3 ± 2.3 vs. 15.5 ± 1.8 years, *p* = 0.32), sex (54.8% vs. 51.4% girls, *p* = 0.53) or baseline AFHC total (11.70 ± 5.23 vs. 11.48 ± 5.06, *p* = 0.71; *n* = 176 non-completers for FAS III and the three baseline scores, because one record had a data-entry misalignment). Completers had higher family affluence (FAS III 10.0 ± 2.7 vs. 8.9 ± 2.6, *p* < 0.001) and lower baseline S-NutLit scores (3.05 ± 1.06 vs. 3.26 ± 0.73, *p* = 0.034). Baseline EDB total scores were lower in completers in the same direction, with the test at the significance threshold (20.99 ± 8.18 vs. 22.62 ± 6.75, *p* = 0.050). All results that follow describe adolescents who finished the session.

Twenty-nine participants (18.7%) were aged 18 or 19 years and gave their own consent. No item-level responses were missing in the final sample at either administration, and no adverse events were reported during the study.

Baseline characteristics overall and by sex are presented in Table 1. The final analytic sample comprised 155 adolescents (70 boys, 45.2%; 85 girls, 54.8%) aged 10–19 years (mean 15.3 ± 2.3 years old). Of the participants, 56 (36.1%) were aged 10–14 years and 99 (63.9%) were aged 15–19 years. According to the FAS III, 18 participants (11.6%) were in the low-affluence category, 42 (27.1%) were in the medium-affluence category and 95 (61.3%) were in the high-affluence category. Boys and girls did not differ in age, FAS III score, or any of the three baseline outcome scores (all *p* ≥ 0.24). Boys had a higher BMI than girls (23.78 vs. 22.24 kg/m^2^, *p* = 0.022, *d* = 0.38) and reported more days of physical activity per week (3.70 vs. 3.14, *p* = 0.039, *d* = 0.34). Mean BMI-for-age z-score was 0.71 (SD 1.18) and was higher in boys than girls (0.96 vs. 0.51, *p* = 0.019). Using WHO cut-offs, 57 participants (36.8%) had overweight, 17 (11.0%) had obesity and 2 (1.3%) had thinness; combining overweight and obesity, 74 participants (47.7%) had excess weight, compared with 25.1% in a national sample of Romanian children aged 5 to 17 years [8]. Twenty-nine participants were aged 18 or 19 years, at or above the upper limit of the WHO reference; restricting the z-score calculation to the 126 participants aged 10 to 17 years gave a similar mean (0.67, SD 1.21) and prevalence of excess weight (45.2%). Weight-management intentions differed by sex (χ^2^ *p* < 0.001): 36.5% of girls and 12.9% of boys were trying to lose weight, whereas 34.3% of boys and 7.1% of girls were trying to gain weight; 31.4% of boys and 29.4% of girls reported doing nothing about their weight. More than half of the sample (54.8%) reported over 4 h of daily recreational screen time, with no difference by sex (*p* = 0.10). Prior participation in nutrition-education activities was reported as none by 68 participants (43.9%), as very little by 69 (44.5%) and as several activities by 18 (11.6%), with no difference by sex (*p* = 0.59).

### 3.2. Module Quiz Performance

Mean total quiz score was 29.5 of 40 (SD 6.3; *n* = 153, since two participants had a missing module score). Mean scores by module were 7.4 (SD 2.4), 6.5 (SD 2.0), 8.0 (SD 1.9) and 7.6 (SD 2.0) of 10 for macro- and micronutrients; water, oxygen and sleep; ultra-processed foods and labels; and kitchen basics, respectively. Using the badge thresholds defined for each module (6–7 bronze, 8 silver, 9–10 gold), the module with the fewest gold badges was water, oxygen and sleep (19.6% gold, 43.8% bronze) and the module with the most was ultra-processed foods and labels (61.4% gold). Total quiz score did not correlate significantly with the change in S-NutLit (*r* = −0.13, *p* = 0.11) or with the change in AFHC total (*r* = −0.13, *p* = 0.12). Quizzes were retakeable and only the most recent attempt was retained for each participant and module.

### 3.3. Internal Consistency of the Measures

Internal consistency was good–excellent for all three scales at both administrations (Table 2). Cronbach’s α was 0.95 (pre) and 0.94 (post) for the S-NutLit, 0.92 and 0.91 for the EDB, and 0.83 and 0.83 for the AFHC; McDonald’s ω ranged from 0.91 to 0.96. The mean inter-item correlation was 0.64 and 0.61 for the S-NutLit, 0.65 and 0.63 for the EDB, and 0.18 and 0.18 for the AFHC.

### 3.4. Change in Outcomes from Pre- to Post-Intervention

**Figure 2:**
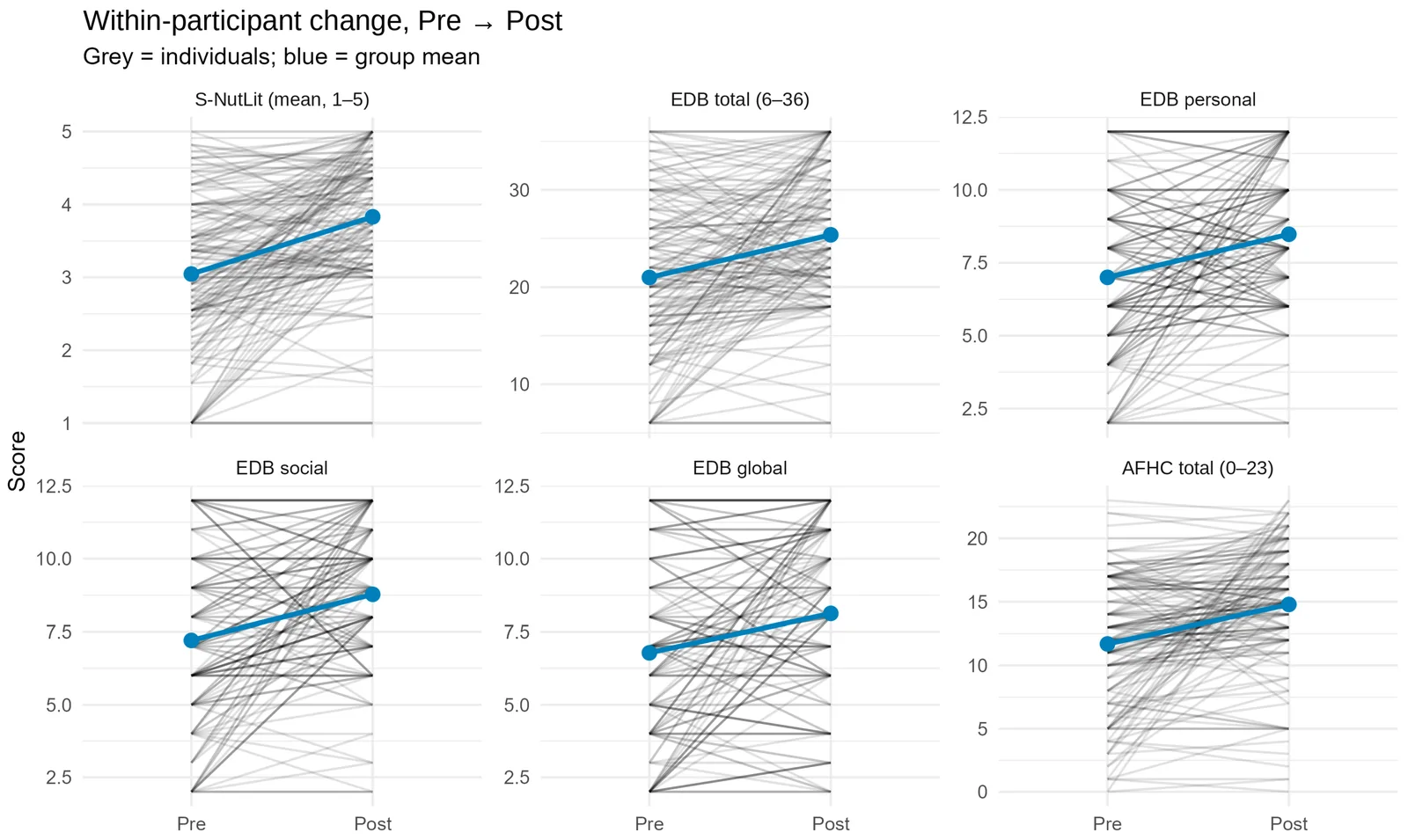
Within-participant change from pre- to post-intervention for each outcome. Grey lines connect individual participants; the blue line marks the group mean.

All six outcome scores increased from pre- to post-intervention (Table 3, Figure 2). The S-NutLit mean rose from 3.05 (SD 1.06) to 3.83 (SD 0.90), a within-person change of 0.78 points (95% CI 0.62–0.95; *t*(154) = 9.14). The EDB total rose from 20.99 (SD 8.18) to 25.37 (SD 7.25), a change of 4.39 points (95% CI 3.06–5.72; *t*(154) = 6.53); the personal facet rose from 7.00 (SD 2.86) to 8.48 (SD 2.57), a change of 1.48 points (95% CI 0.98–1.97); the social facet rose from 7.21 (SD 2.99) to 8.77 (SD 2.55), a change of 1.57 points (95% CI 1.06–2.08); and the global facet rose from 6.78 (SD 3.19) to 8.12 (SD 3.04), a change of 1.34 points (95% CI 0.83–1.85). The AFHC total rose from 11.70 (SD 5.23) to 14.79 (SD 4.93), a change of 3.09 points (95% CI 2.21–3.97; *t*(154) = 6.93). All six changes remained significant after Benjamini–Hochberg correction (all p_BH < 0.001). Cohen’s *dz* was 0.73 (95% CI 0.56–0.91) for the S-NutLit, 0.52 (95% CI 0.36–0.69) for the EDB total, 0.47 (95% CI 0.31–0.64), 0.49 (95% CI 0.32–0.65) and 0.42 (95% CI 0.25–0.58) for the EDB personal, social and global facets, and 0.56 (95% CI 0.39–0.73) for the AFHC total. Difference scores departed from normality for every outcome (Shapiro–Wilk *p* ≤ 0.0002); the Wilcoxon signed-rank test confirmed all six changes (all p_BH < 0.001), with matched-pairs rank-biserial correlations of 0.74 for S-NutLit, 0.56 for EDB total, 0.53 for EDB personal, 0.57 for EDB social, 0.47 for EDB global, and 0.65 for AFHC total.

### 3.5. Item-Level Change on the Adolescent Food Habits Checklist

Of the 23 AFHC items, 17 shifted significantly toward the healthier response after Benjamini–Hochberg correction, and none shifted in the unhealthy direction (Table S6). The largest shifts were observed for usually avoiding fried foods (34.8% to 62.6% healthy responses, p_BH < 0.001), avoiding sausages and burgers (45.8% to 68.4%, p_BH < 0.001) and trying to eat plenty of fruit and vegetables (57.4% to 76.1%, p_BH < 0.001). Among the five reverse-worded items, three changed significantly (item 3, 32.3% to 51.0%, p_BH = 0.001; item 21, 60.0% to 72.3%, p_BH = 0.023; item 8, 45.2% to 56.1%, p_BH = 0.046) and two did not (items 14 and 18). The remaining non-significant items were item 23 (generally tries to have a healthy diet; 74.2% to 81.3%, p_BH = 0.140), item 1 (low-fat option for lunch away from home; 38.1% to 43.9%, p_BH = 0.369), item 19 (fruit for between-meal snacks; 60.6% to 64.5%, p_BH = 0.544) and item 17 (spreads butter/margarine thinly; 61.9% to 63.2%, p_BH = 0.883).

### 3.6. Moderation by Sex, Age Group and Family Affluence

None of the time × moderator interactions was significant for any outcome; raw *p*-values ranged from 0.13 to 0.97, and all remained non-significant after Benjamini–Hochberg correction (p_BH ≥ 0.90; Table S7). Boys and girls showed significant increases on every outcome when tested separately (all simple-contrast *p* ≤ 0.018; Table S8), and the sex difference in the size of the change was not significant for any outcome (*p* ≥ 0.13; Table S7). The change in S-NutLit was 0.79 points in boys and 0.78 in girls; in EDB total, 3.84 and 4.84; in AFHC total, 3.41 and 2.82. Both age bands showed significant increases on every outcome (all 12 simple contrasts *p* ≤ 0.001; Table S8). Medium- and high-affluence participants showed significant increases on every outcome (all *p* ≤ 0.003; Table S8). Low-affluence participants (*n* = 18) showed significant increases on the S-NutLit (change 0.55, *p* = 0.032) and the AFHC (change 4.56, *p* < 0.001), whereas their changes on the EDB total (2.28, *p* = 0.25) and on the personal (0.56, *p* = 0.45), social (0.78, *p* = 0.31) and global (0.94, *p* = 0.21) facets were not significant. These subgroup analyses were exploratory, and the estimates for the low-affluence group are imprecise.

### 3.7. Predictors of Post-Intervention Scores

In the ANCOVA-style regression models (Table S9), the baseline score was the strongest predictor of the post-intervention score for every outcome (unstandardised β = 0.23–0.44, all *p* ≤ 0.002). Higher FAS III score independently predicted higher post-intervention S-NutLit (β = 0.081 per point, *p* = 0.001), EDB total (β = 0.47, *p* = 0.025), EDB personal (β = 0.23, *p* = 0.002) and EDB social (β = 0.17, *p* = 0.031). Older age predicted lower post-intervention EDB total (β = −0.57 per year, *p* = 0.016) and EDB global (β = −0.31, *p* = 0.002). More physical-activity days predicted higher post-intervention S-NutLit (β = 0.093 per day, *p* = 0.026). Sex (all *p* ≥ 0.11) and prior nutrition-education exposure (all *p* ≥ 0.14) were not significant predictors of any post-intervention score. The models explained 16.8% (EDB social) to 29.2% (EDB global) of the variance in post-intervention scores (adjusted R^2^ = 12.9–25.8%).

## 4. Discussion

The results represent immediate within-participant changes after one session. Without a concurrent control group, and with the post-test administered directly after the session, these changes cannot be attributed to the platform. Completing the same questionnaire twice in a single sitting may itself alter responses [35]. An immediate post-test also encourages respondents to answer in the direction they perceive as expected, through social desirability and demand characteristics (see AFHC item 16, Section 4.2). The AFHC items with the largest changes (fried food, sausages and burgers, fruit and vegetables) refer to food categories presented in the modules minutes earlier, and their change may reflect recall of that content. Because participants were not selected on their baseline scores, regression to the mean does not alter the expected sample mean; it does, however, affect individual change. The baseline coefficients in the regression models were well below 1 (β 0.23 to 0.44), indicating larger gains among participants with low initial scores, which is relevant when subgroups with different baseline levels are compared. AFHC scores record reported food choices; at an immediate post-test, they most likely reflect short-term intentions and cannot show whether dietary intake changed. The evidence that the curriculum can be delivered and completed in a fully automated format, and the estimates of short-term change needed to plan a controlled trial, are not affected by these limits.

### 4.1. Nutrition Literacy

After one session with the AHOPE platform, the S-NutLit mean was 3.83 on a 1 to 5 scale, against 3.05 before the session, a within-person change of 0.78 points. A meta-analysis of six randomised trials of gamified nutrition interventions in children and adolescents estimated a standardised mean difference of 0.88 (95% CI 0.05 to 1.75) for nutritional-knowledge scores [21]. That estimate compares gamified and control groups, while our *dz* describes change within a single group, so the two figures cannot be compared directly. Single studies report gains of similar size. In a Mexican pilot with children aged 8 to 10 years, the video game FoodRateMaster increased food knowledge from 56.9 to 67.8 points after gameplay [36]. In an Italian food-literacy programme, the nutrition-literacy score of adolescents rose from 49.1 to 56.1 immediately after the intervention and was 54.4 three months later [37]. In a Warsaw primary school, in children younger than the present sample, a one-time nutrition-education session raised the nutrition-knowledge score from 18.1 to 21.1 points, and the score was still 19.7 six months later [38]. The two European programmes relied on an instructor to deliver the content, and the Mexican video game was evaluated in children younger than the present sample; here, a change of the same order followed a session in which the platform alone carried the teaching, with adult involvement limited, at most, to supervision.

The pilot study behind the AHOPE curriculum [16] is the closest comparison, because it delivered the same content through a classroom teacher, so the two studies differ mainly in delivery format. In that pilot, 127 fifth-grade pupils improved in reading food labels, understanding dietary recommendations, resisting unhealthy food choices and willingness to try new vegetables. A similar pattern of short-term change was observed here without an instructor, although the two studies used different outcome measures and neither had a concurrent control group. Across nutrition-education programmes for schoolchildren, the size of the improvement was unrelated to the number of tools, lesson duration or programme length [17], and instructor-led delivery does not prevent decay: in the Polish ABC of Healthy Eating programme, 319 pupils aged 11 to 12 years had gained 1.8 points more in nutrition knowledge than 145 controls at three months but retained only 0.4 points at nine months [39]. The platform was built around self-determination theory, with quizzes, missions and rewards intended to support the need for competence and autonomy [19], and badges have been shown experimentally to raise perceived competence [40]; whether these features contributed to the change observed here cannot be established in a single-arm study. Because the outcome measures also differed from those of the pilot, the two delivery formats cannot be compared on effectiveness.

If the change reflected learning of the module content, pupils who answered more quiz items correctly would be expected to show larger changes. Total quiz score was not associated with change in the S-NutLit (*r* = −0.13, *p* = 0.11) or in the AFHC total (*r* = −0.13, *p* = 0.12), and both coefficients were slightly negative. This pattern is more compatible with change driven by exposure to the session, repeated testing or responding in the expected direction than with change driven by mastery of the content. Two features of the data limit this interpretation. Quizzes could be retaken and only the final score was retained, so for the 21, 29, 11 and 18 participants whose first and final scores differed in Modules 1 to 4, the final score may not reflect first-attempt mastery. The quizzes also tested knowledge of the module content, whereas the S-NutLit measures self-perceived skills and the AFHC reported food choices, and a weak association could partly reflect the difference between these constructs. The correlation therefore supports an exposure or demand explanation without excluding the alternatives.

### 4.2. Engagement in Dietary Behaviour and Food Habits

The EDB total increased by 4.39 points and the AFHC total by 3.09 points. For these two behavioural scores, the literature is less favourable than for knowledge. The most recent meta-analysis of serious games, pooling 20 randomised trials, found an effect on physical activity (SMD 0.61) and no effect on dietary change [22]. A scoping review of 41 digital dietary interventions for children and adolescents found favourable results on all outcomes in 15% of experimental studies, mixed results in 58% and none in 28% [41]. The changes observed here are larger than the between-group effects in these reviews, as expected for an uncontrolled pre–post comparison that also captures repeated testing and socially desirable responding. The AFHC records self-reported everyday choices, not measured intake, and the second administration followed the session immediately, so the difference is compatible with a short-term shift in intentions without any change in behaviour [42]. Two uncontrolled pre and post studies with longer exposure give a reference point. The Food Game programme improved the dietary score of 184 Italian adolescents from 7.6 to 8.2 over one academic year [24]. A digital nudge in a smart school canteen increased the frequency of fruit consumption and reduced that of sweets and sugary drinks among 502 Chinese adolescents over three months [43]. The present study adds that a self-reported shift of comparable size is measurable after one session; it does not show that the shift persists. To our knowledge, no minimally important difference has been published for the S-NutLit, the EDB or the AFHC. The changes reported here are described by their statistical size only, and these data cannot show whether a change of this size corresponds to a meaningful difference in nutrition skills, dietary engagement or food choices.

At item level, the largest changes were on avoiding fried foods (34.8% to 62.6% healthy responses), avoiding sausages and burgers (45.8% to 68.4%) and trying to eat plenty of fruit and vegetables (57.4% to 76.1%), all of which name a food category covered by the curriculum. Two of the five reverse-worded items, eating sweet snacks between meals and including chocolate or biscuits in a packed lunch, did not change. Both describe snacking outside meals, the behaviour most closely tied to adolescents’ growing autonomy over what they eat [5], and neither item changed measurably after the session. A similar gap between knowledge and practice has been reported in the region, since, among 1440 Polish students aged 10 to 18 years, the mean score was 51% for nutrition-related knowledge and 32% for nutrition-related practice [44], and a systematic review of 13 studies in children and adolescents found the association between nutrition knowledge and dietary behaviour inconsistent, with 4 studies reporting none [13].

Two item-level results point to a divergence between the curriculum and the checklist, which complicates the interpretation of change on those items. First, AFHC item 16 scores choosing a diet soft drink as the healthier response, and healthy responses on this item rose from 49.0% to 65.8% (p_BH = 0.002), although Module 3 presents diet drinks sweetened with aspartame or sucralose as ultra-processed and not necessarily healthier than the sugar-sweetened version. Pupils who had just been taught that diet drinks are not a healthier choice reported choosing them more often, which suggests that part of the item-level change reflects a general tendency to answer in the direction perceived as approved rather than a specific uptake of the taught content [45]. Second, the AFHC was developed in 2002 and validated against dietary fat and fibre intake [28], and five of its items (items 1, 5, 6, 11 and 17) score a low-fat choice as the healthier response. The AHOPE curriculum instead distinguishes unsaturated from saturated and trans fats and does not present total fat reduction as a goal. Two of the four remaining items that did not shift are low-fat items (item 17, spreading butter or margarine thinly, 61.9% to 63.2%; item 1, a low-fat option for lunch away from home, 38.1% to 43.9%), whereas the other three low-fat items did shift; the pattern is consistent with a divergence between the instrument and the curriculum on this content domain. Both observations indicate that the AFHC, in its original form, is only partly aligned with current nutrition guidance and with the AHOPE content, and that item-level change on it should be read as a change in reported choices measured against the 2002 scoring key.

The forced-choice format used on the AHOPE platform further limits what the AFHC scores can show. Ten items of the original checklist (items 1, 6, 7, 11, 16, 17, 18, 19, 20 and 21) presuppose a situation that not every adolescent experiences, such as having lunch away from home (item 1), buying crisps (item 6), buying soft drinks (item 16) or taking a packed lunch (item 18), and offer a “not applicable” response for respondents without that experience [28]. In the adapted version, these respondents had to answer true or false, which may introduce responses unrelated to actual behaviour and reduce the stability of answers on these items between administrations. Because the same format was used at both time points, the forced choice affects the absolute level of the scores and their comparability with other AFHC studies more than it affects the within-person comparison. Together with the divergence of the 2002 scoring key from current guidance on fat quality, described above, this means that the AFHC total in this study is an index of reported food choices scored against the original key; its validity as a measure of diet quality in the adapted format has not been tested.

Internal consistency was lower for the AFHC (α = 0.83) than for the S-NutLit (α ≥ 0.94) and the EDB (α ≥ 0.91). The AFHC is a true/false checklist of 23 distinct behaviours, and its mean inter-item correlation of 0.18 is the value expected for such an instrument [28]; the S-NutLit and EDB items measure one construct each with 5- and 6-point response formats [14,27].

### 4.3. Sex, Age and Family Affluence

The interaction tests gave no evidence that change differed by sex, age band or family affluence. These tests had limited power, especially for the low-affluence group (*n* = 18), and they do not show that change was equivalent across subgroups. A meta-analysis of 16 randomised trials of gamified physical-activity interventions (2407 participants) found no interaction with age, gender or BMI and no difference between adolescents and adults [46]. The Polish ABC of Healthy Eating programme produced gains of similar size in girls and boys and in low- and higher-affluence pupils at three months [39]. The absence of a detectable sex difference is of interest because weight-related intentions differed strongly by sex: 36.5% of girls and 12.9% of boys were trying to lose weight, and 34.3% of boys and 7.1% of girls were trying to gain weight. Among adolescents from the same region, 26.5% scored above the EAT-26 clinical cut-off for eating-disorder risk, with girls having 1.61 times the odds of boys [47], and close to 80% of parents reported discussing weight with their child at least sometimes [48].

Among the 18 low-affluence pupils, the S-NutLit increased by 0.55 points (*p* = 0.032) and the AFHC total by 4.56 points (*p* < 0.001), whereas the changes on the four engagement scores were not statistically significant; the EDB total changed by 2.28 points (*p* = 0.25), compared with 5.02 and 4.51 points in the medium- and high-affluence groups. With 18 participants, these estimates are imprecise. The non-significant engagement changes cannot be taken as evidence that engagement remained unchanged in this group, and the interaction tests gave no evidence that change differed from the other affluence groups. In the regression models, higher affluence predicted higher post-intervention literacy and engagement after adjustment for baseline, whereas sex did not. Among 968 Canadian adolescents, the nutrition-knowledge score rose by 0.52 percentage points for every CAD 10,000 of median neighbourhood family income, and sex was not associated with any knowledge score in the adjusted models [49]. Adolescents from lower-affluence families across 36 European countries eat less fruit and vegetables and show a less favourable trend over time [50]. The only scoping review of digital dietary interventions for people with a lower socioeconomic position (17 studies) found no conclusive evidence for any single behaviour-change technique; social support was rare but appeared more often in effective interventions [51]. In the Polish programme, no subgroup difference by sex or affluence remained significant at nine months, and the authors concluded that family affluence did not shape the long-term maintenance of knowledge [39]. A follow-up study therefore needs enough low-affluence participants to test this subgroup directly.

### 4.4. Delivery Format and Durability

For the AHOPE project, the relevant finding is that the curriculum could be delivered and completed in a fully automated format, and that completion was followed by short-term changes in the same direction as those reported for the teacher-delivered version [16]. School-based nutrition education has been limited by the trained staff it requires. Adolescents who used a food, nutrition or health app scored 2.55 percentage points higher on nutrition knowledge than non-users in the Canadian sample, yet most such apps target weight loss and intake tracking rather than food literacy [49]. A systematic review of 16 randomised trials in 31,971 adolescents reported adherence rates of 63% to 85.5% and improvements in dietary habits for the interventions that used personalised feedback (nine trials) and gamification (one trial of 36 participants). The same review identified long-term engagement as the unresolved problem of digital dietary interventions [52]. AHOPE has both features; the present data say nothing about engagement beyond one session, and the evidence on gamification in this age group rests on very few trials. A structured review of evaluated childhood obesity prevention programmes in Europe between 2015 and 2024 reached the same conclusion at a wider scale: interventions consistently improved intermediate outcomes such as knowledge and selected dietary behaviours far more than anthropometric endpoints, and short-duration, single-setting educational interventions had the smallest and least consistent effects on weight [53]. The present study is an intervention of exactly that kind, and its results should be read as evidence about intermediate outcomes only.

Across gamified physical-activity interventions, the effect was still detectable at follow-ups averaging 14 weeks after the intervention ended (g = 0.15, 95% CI 0.07 to 0.23), which does not support a novelty effect [46]. For standalone digital interventions, a post-intervention effect of g = 0.14 was no longer significant at the longest follow-up [54]. At umbrella level, across 440 randomised trials of eHealth and mHealth interventions in children and adolescents, dietary improvements were modest overall, shorter interventions produced larger physical-activity effects than longer ones, and anthropometric change favoured longer programmes [55]. The Warsaw one-time session in younger children retained most of its knowledge gain at six months [38], and the Italian food-literacy programme retained most of its gain at three months [37], whereas the advantage of the Polish programme over controls fell from 1.8 to 0.4 points between three and nine months as the control group caught up through regular schooling [39]. A waitlist- or usual-practice-controlled trial with a follow-up of several weeks would show how much of the observed change can be attributed to the platform and whether it persists. Recording the interval between administrations, session duration and platform usage would also address a documented weakness of this literature, in which intervention characteristics are incompletely reported [17]. The low-affluence subgroup should be oversampled, and the platform’s behaviour-change techniques catalogued against the taxonomy used for that group, in which social support appeared more often in effective than in ineffective interventions [51]. A version of the platform that addresses weight attitudes and weight-related communication could then be tested in boys and girls separately [47,48]. A longer follow-up could also test whether short-term changes in reported food choices during adolescence attenuate the compulsive-eating and soft-drink patterns associated with higher BMI in Romanian young adults [10].

### 4.5. Strengths and Limitations

The achieved sample of 155 paired observations is large for a single-arm school study and gives 80% power to detect a within-person effect of *dz* = 0.23, well below the effects observed; its sex distribution and age range match the population the platform targets. Participants came from 74 schools across 19 cities, which limits the influence of any single school or region on the results. The three instruments had high internal consistency at both administrations (Table 2), the false-discovery-rate correction was applied across outcomes and across the 23 AFHC items, and parametric and non-parametric tests agreed on every outcome. The study provides data on a gamified nutrition-education platform from Eastern Europe, a region not represented in the meta-analyses in this field [21,22].

Fewer than half of the adolescents who began the session (46.7%) completed it. Completers came from more affluent families and started with lower nutrition-literacy scores than those who stopped; their baseline dietary-engagement scores were lower in the same direction, at the significance threshold. The results therefore describe a selected group and may not apply to adolescents who did not finish, who were, on average, less affluent. Lower starting scores leave more room for an increase, so the change might have been smaller among adolescents with higher baseline scores, such as those who stopped, whose scores were higher on average. Reasons for non-completion were not recorded.

These results describe the change that follows one session under a single-arm design with an immediate post-test. The share of that change attributable to repeated testing [56] and its persistence are the questions a controlled trial with delayed follow-up answers. The platform requires each pupil to complete the four modules and their quizzes between the pre- and post-questionnaire, but the data export does not record submission times, so the interval between the two administrations, and therefore the actual time spent on the modules, is unknown for every participant. The four modules were designed to take 7 to 8 min each, but the data cannot confirm that any participant spent that long, and a pupil who clicked through the quizzes quickly and a pupil who read carefully are indistinguishable in these data. Height and weight were self-reported, as in school surveys of this size [57], and BMI served only as a descriptive covariate. The Romanian versions of the three scales were translated and back-translated but were not tested in cognitive interviews or a pilot study, and their factor structure was not examined in this sample. High internal consistency indicates that respondents answered the items of each scale consistently, which is insufficient to establish that the items measured the intended constructs in Romanian adolescents. The concern applies most to the S-NutLit, developed for young adults [14], since the youngest participants here were 10 years old. Younger pupils may have interpreted some items differently from older ones, so differences between age bands may partly reflect item interpretation. Cognitive interviews and a confirmatory factor analysis across the adolescent age range are needed before these versions are used in a controlled trial.

Recruitment through established school partnerships and online parents’ groups is likely to have favoured digitally connected families, consistent with the predominance of the high-affluence category in the sample (61.3%). Outcomes were self-reported and none was assessed by a blinded observer. Socially desirable responding is a particular concern for the AFHC. The items with the largest changes named foods covered in the modules, the forced true/false format required respondents without the relevant experience to choose an answer, possibly the one they perceived as approved, and item 16 shifted toward the response the curriculum had questioned. Because the post-test followed the session immediately, AFHC change reflects reported intentions and provides no measure of dietary behaviour. The low-affluence subgroup comprised 18 participants, which is too few for a precise estimate of change in this subgroup. With a median of two participants per school across 74 schools, school-level effects could not be estimated separately from participant-level variance. The study was not registered in a public trial registry; the protocol was approved by the Research Ethics Subcommittee of Babeș-Bolyai University before enrolment (approval No. 4299/25.03.2026; request No. 143/11.03.2026).

## 5. Conclusions

In Romanian adolescents aged 10 to 19 years who completed one session with the AHOPE gamified platform, nutrition-literacy and dietary-engagement scores were higher immediately after the session than before it, and responses on an adapted food-habit checklist shifted toward the healthier option. These findings are immediate within-participant changes in self-reported measures. Fewer than half of those who began the session (46.7%) completed it, and completers came from more affluent families, so the results may not apply to adolescents who stopped. The single-arm design and immediate post-test do not allow conclusions about whether the platform caused these changes, whether they persist or whether adolescents changed what they eat. The study demonstrates that the curriculum can be delivered and completed in a fully automated format without an instructor. Testing effectiveness requires a controlled trial with delayed follow-up, repeated dietary assessment and a larger low-affluence subgroup.

## Figures and Tables

**Table 1:** Participant characteristics, overall and by sex (*N* = 155).

Characteristic	Overall	Boys (*n* = 70)	Girls (*n* = 85)	*p*
Age, years	15.31 (2.28)	15.53 (2.39)	15.13 (2.19)	0.284
BMI, kg/m^2^	22.94 (4.13)	23.78 (4.26)	22.24 (3.90)	0.022
BMI-for-age z-score	0.71 (1.18)	0.96 (1.22)	0.51 (1.11)	0.019
FAS III score (0–13)	10.01 (2.70)	10.29 (2.74)	9.78 (2.67)	0.245
Physical activity, days/week	3.39 (1.65)	3.70 (1.80)	3.14 (1.48)	0.039
S-NutLit, baseline (1–5)	3.05 (1.06)	3.13 (1.10)	2.98 (1.03)	0.376
EDB total, baseline (6–36)	20.99 (8.18)	21.11 (8.33)	20.88 (8.10)	0.862
AFHC total, baseline (0–23)	11.70 (5.23)	11.89 (5.60)	11.54 (4.93)	0.688
Age group, *n*	—	—	—	0.791
10–14 y	56	24	32	
15–19 y	99	46	53	
FAS category, *n*	—	—	—	0.998
Low	18	8	10	
Medium	42	19	23	
High	95	43	52	
Weight-management goal, *n*	—	—	—	<0.001
Trying to gain weight	30	24	6	
Trying to stay the same	38	15	23	
Trying to lose weight	40	9	31	
Doing nothing about it	47	22	25	
Recreational screen time, *n*	—	—	—	0.101
Less than 1 h/day	2	0	2	
1 to 2 h/day	23	7	16	
2 to 4 h/day	45	18	27	
More than 4 h/day	85	45	40	
Prior nutrition-education activities, *n*	—	—	—	0.588
None	68	31	37	
Very little	69	29	40	
Several activities	18	10	8	

Note: Values are mean (SD) for continuous variables and *n* for categorical variables. *p*-values are from Welch’s *t*-test (continuous) or the chi-squared test (categorical). FAS III, Family Affluence Scale III; S-NutLit, Short Nutrition Literacy Scale; EDB, Engagement in Dietary Behaviour scale; AFHC, Adolescent Food Habits Checklist. AFHC scores were obtained with an adapted forced-choice version without the original “not applicable” option and are not directly comparable with AFHC scores reported in other studies.

**Table 2:** Internal consistency of the three outcome scales at both administrations.

Scale	*k*	α	ω Total	Mean *r*	Wave
S-NutLit	11	0.951	0.963	0.637	Pre
	11	0.943	0.959	0.605	Post
EDB	6	0.919	0.936	0.652	Pre
	6	0.907	0.933	0.633	Post
AFHC	23	0.833	0.910	0.178	Pre
	23	0.826	0.908	0.175	Post

Note: *k*, number of items; α, Cronbach’s alpha; ω, McDonald’s omega; Mean *r*, mean inter-item correlation.

**Table 3:** Change in outcome scores from pre- to post-intervention (paired analysis, *N* = 155).

Outcome	Pre, M (SD)	Post, M (SD)	Δ [95% CI]	*t*(154)	p_BH	*dz*
S-NutLit (1–5)	3.05 (1.06)	3.83 (0.90)	0.78 [0.62, 0.95]	9.14	<0.001	0.73
EDB total (6–36)	20.99 (8.18)	25.37 (7.25)	4.39 [3.06, 5.72]	6.53	<0.001	0.52
EDB personal	7.00 (2.86)	8.48 (2.57)	1.48 [0.98, 1.97]	5.90	<0.001	0.47
EDB social	7.21 (2.99)	8.77 (2.55)	1.57 [1.06, 2.08]	6.09	<0.001	0.49
EDB global	6.78 (3.19)	8.12 (3.04)	1.34 [0.83, 1.85]	5.22	<0.001	0.42
AFHC total (0–23)	11.70 (5.23)	14.79 (4.93)	3.09 [2.21, 3.97]	6.93	<0.001	0.56

Note: Δ, mean within-person change (post − pre); p_BH, paired *t*-test *p*-value after Benjamini–Hochberg correction across the six outcomes; *dz*, Cohen’s *dz* for paired data. All Wilcoxon signed-rank p_BH < 0.001 (not tabulated). AFHC scores were obtained with an adapted forced-choice version without the original “not applicable” option and are not directly comparable with AFHC scores reported in other studies.

## Data Availability

The data supporting this study’s findings are held by Babeș-Bolyai University; access to the data and to the R analysis code can be requested from the corresponding author upon reasonable request, with the permission of Babeș-Bolyai University. Public deposition of the individual-level dataset is not possible because most participants were minors and the informed consent obtained (ethics approval No. 4299/25.03.2026, Research Ethics Subcommittee of Babeș-Bolyai University) does not extend to public sharing of individual records. Requests are honoured subject to the consent terms and Regulation (EU) 2016/679 (GDPR).

## Supplementary Materials

The following supporting information can be downloaded at: https://www.mdpi.com/article/10.3390/nu18203447/s1. Table S1: TREND checklist for this non-randomised evaluation; Tables S2–S5: Detailed description of the AHOPE session content (module structure and topic coverage; end-of-module quiz question stems; module-level badges and score thresholds; final session-level badges); Table S6: Item-level change on the Adolescent Food Habits Checklist (AFHC), ranked by significance; Table S7: Sex differences in the size of the pre-to-post change, and time × moderator interaction tests; Table S8: Simple contrasts of the pre-to-post change within levels of sex, age band and family affluence; Table S9: ANCOVA-adjusted predictors of post-intervention scores; Table S10: Baseline characteristics of participants who completed the session and those who did not.

## Abbreviations

AFHC, Adolescent Food Habits Checklist; AHOPE, Adolescents’ Healthy Options and Positive Eating; ANCOVA, analysis of covariance; BMI, body mass index; CI, confidence interval; *dz*, Cohen’s *dz* (paired effect size); EAT-26, Eating Attitudes Test, 26 items; EDB, Engagement in Dietary Behaviour scale; FAS III, Family Affluence Scale III; GDPR, General Data Protection Regulation; p_BH, *p*-value after Benjamini–Hochberg false-discovery-rate correction; SD, standard deviation; SMD, standardised mean difference; S-NutLit, Short Nutrition Literacy Scale; TREND, Transparent Reporting of Evaluations with Nonrandomized Designs; WHO, World Health Organization; α, Cronbach’s alpha; β, unstandardised regression coefficient; ω, McDonald’s omega; χ^2^, chi-squared statistic.
