# Supplementary material for: Short-Term Changes in Nutrition Literacy, Dietary Engagement, and Reported Food Habits Among Adolescents After One Session with a Gamified Digital Nutrition-Education Platform: A Single-Arm Pre–Post Study
Source: Nutrients. 2026 Oct 20;18(20):3447. doi: 10.3390/nu18203447 (PMC13708113; doi:10.3390/nu18203447)
Supplement: Supplementary file 1 [file nutrients-18-03447-s001.zip › nutrients-4598413-supplementary.pdf]

Short-Term Changes in Nutrition Literacy, Dietary Engagement, and Reported Food Habits Among Adolescents After One Session with a Gamified Digital Nutrition Education Platform: A Single-Arm Pre-Post Study

Supplementary Materials

Single-group pre–post (non-randomised) evaluation of one session with the AHOPE gamified digital platform; analytic sample N = 155. Reporting follows the TREND checklist; items that presuppose randomised allocation to multiple study conditions are marked as not applicable.

Table S1. TREND (Transparent Reporting of Evaluations with Nonrandomized Designs) Statement checklist: item-by-item documentation of how each of the 22 reporting items is addressed in this single-arm pre-post study.

Section / topic	Item	Checklist item (official descriptor)	AHOPE study – as reported
**Title & abstract**	1	How units were allocated; structured abstract; target population/sample	The title identifies a single-arm pre-post study (no allocation); a structured abstract (Background/Objectives, Methods, Results, Conclusions) is provided. Target population: Romanian adolescents aged 10–19; 332 individuals began the study, analytic sample N = 155. Location in manuscript: Title; Abstract.
**Introduction – Background**	2	Scientific background and rationale; theories used in designing the intervention	Rising adolescent overweight and poor diet in Europe and Romania; the inconsistent knowledge–behaviour association motivating literacy-plus-engagement targets; gamification rationale grounded in self-determination theory (competence, autonomy, relatedness). Location in manuscript: Introduction.
**Methods – Participants**	3	Eligibility (incl. levels); recruitment method and sampling; recruitment setting; locations	Eligibility: pupils aged 10–19 enrolled in Romanian schools with access to a digital device; exclusions: declined consent, language barrier, severe cognitive disability. Recruitment: partner schools in Cluj-Napoca, extended nationally through teachers' networks and online parents' groups (self-selected convenience sample), under the same approved consent procedure. Settings: classrooms under teacher supervision during the school term, or outside school during the summer holiday. Location in manuscript: Methods (2.1, 2.2).
**Interventions**	4	Content; delivery method; unit of delivery; deliverer; setting; exposure quantity and duration; time span; adherence activities	Content: four modules (Macro- and Micronutrients; Water, Oxygen and Sleep; Ultra-Processed Foods and Labels; Kitchen Basics) converting a validated teacher-delivered curriculum. Delivery: fully automated gamified platform on personal smartphones; deliverer: the platform itself, no instructor; unit of delivery: the individual. Exposure: one session, four modules of 7–8 min each, each ending in a retakeable 10-item quiz with instant feedback; badges (bronze/silver/gold; overall badge of 40) as engagement features; the post-questionnaire unlocked only after all four quizzes. Location in manuscript: Methods (2.1, 2.4); Supplementary Tables S2–S5.

Section / topic	Item	Checklist item (official descriptor)	AHOPE study – as reported
**Objectives**	5	Specific objectives and hypotheses	Pilot pre–post evaluation describing short-term change in nutrition literacy, dietary engagement and reported food choices from immediately before to immediately after one session, and exploring whether this change differed by sex, age band and family affluence; not designed to test effectiveness; no comparator hypothesis (single-group design). Location in manuscript: Introduction (final paragraph); Methods (2.1).
**Outcomes**	6	Clearly defined primary and secondary outcomes; data-collection methods; validated-instrument information	Six outcome scores: S-NutLit total (11 items), EDB total and personal/social/global facets (6 items), adapted AFHC total (23 items, five reverse-scored, forced binary format without the original 'not applicable' option; scores not directly comparable with the original checklist). Self-administered within the platform, approximately 20 minutes; internal consistency in this sample: α 0.83–0.95, ω 0.91–0.96 at both administrations. Romanian versions translated and back-translated; no cognitive interviews, pilot testing or factor analysis were conducted (acknowledged as a limitation). Location in manuscript: Methods (2.5); Results (3.3, Table 2); Discussion (4.5).
**Sample size**	7	How sample size was determined; interim analyses and stopping rules	No a priori calculation; the collection window determined the achieved sample. Sensitivity analysis: N = 155 gives 80% power for dz = 0.23 (α = 0.05, two-sided); no interim analyses or stopping rules. Location in manuscript: Methods (2.6).
**Assignment method**	8	Unit and method of assignment; bias minimisation for non-randomisation	Not applicable – single-group design, no allocation to study conditions. Location in manuscript: Methods (2.1).
**Blinding (masking)**	9	Whether participants, deliverers and outcome assessors were blinded	Not applicable / not feasible – an open, self-report educational intervention; all outcomes self-reported by participants. Location in manuscript: Methods (2.1, 2.5); Discussion (4.5).
**Unit of analysis**	10	Smallest unit analysed; analytic method if it differs from the unit of assignment	The individual adolescent. With a median of two participants per school across 74 schools, school-level effects could not be modelled separately from participant-level variance; acknowledged as a limitation. Location in manuscript: Methods (2.6); Discussion (4.5).
**Statistical methods**	11	Methods to compare groups; additional analyses; imputation; software	Paired t-tests confirmed by Wilcoxon signed-rank; Cohen's dz with 95% CI and matched-pairs rank-biserial correlations; McNemar's test for AFHC items; Benjamini-Hochberg false discovery rate correction in three families (6 outcomes, 23 items, 18 interactions); linear mixed-effects models (random intercept, Satterthwaite degrees of freedom) for moderation; ANCOVA-style regression for predictors; no imputation required (no missing item-level data in the analytic sample); R version 4.6.1. Location in manuscript: Methods (2.6).

Section / topic	Item	Checklist item (official descriptor)	AHOPE study – as reported
**Results – Participant flow**	12	Flow of participants through each stage (diagram strongly recommended); protocol deviations	333 pre-questionnaire records → 332 individuals (one duplicate code; earlier record retained) → 155 completed the post-questionnaire and were analysed; the 177 non-completers reflect incomplete exposure (the post-questionnaire unlocks only after all four quizzes), not loss to follow-up; flow diagram provided. Deviation from the initial plan: recruitment extended beyond the Cluj-Napoca partner schools under the same approved consent procedure. Location in manuscript: Results (3.1, Figure 1); Methods (2.2).
**Recruitment**	13	Dates defining the periods of recruitment and follow-up	Data collected between 31 March and 19 August 2026; pre- and post-questionnaires administered within the same session, no delayed follow-up. Location in manuscript: Methods (2.1).
**Baseline data**	14	Baseline characteristics; lost-vs-retained comparison; study population vs target population	Table 1 reports characteristics overall and by sex; completers and non-completers compared on the pre-questionnaire variables (Table S10; completers more affluent, with lower baseline S-NutLit scores and a borderline difference in baseline EDB scores); sample weight status contrasted with a national Romanian sample. Location in manuscript: Results (3.1, Table 1); Table S10.
**Baseline equivalence**	15	Data on group equivalence at baseline; statistical methods used to control for baseline differences	Not applicable in the between-condition sense (single group); the completer vs non-completer comparison serves as the attrition/selection check. Location in manuscript: Results (3.1; Table S10); Discussion (4.5).
**Numbers analysed**	16	Denominator per analysis; whether the analysis was intention-to-treat	N = 155 for all six outcomes (no missing item-level data at either administration); module quiz analyses n = 153 (two missing module scores); complete-case throughout. Location in manuscript: Results (3.1, 3.2); Tables 1 and 3.
**Outcomes and estimation**	17	Summary of results, effect size and confidence interval per outcome; inclusion of null findings	Table 3 reports pre/post means (SD), within-person change with 95% CI, t statistic, p_BH and dz with 95% CI for all six outcomes; rank-biserial correlations reported; item-level shifts in Table S6. Null findings reported in full: no significant time × moderator interaction, non-significant engagement changes in the low-affluence subgroup (n = 18; imprecise estimates), non-significant quiz-score correlations. Location in manuscript: Results (3.4–3.7; Tables 3, S6–S9; Figure 2).
**Ancillary analyses**	18	Summary of other analyses, indicating which are pre-specified or exploratory	Primary: pre–post change in the six outcome scores. Secondary: AFHC item-level McNemar tests. Exploratory: moderation by sex, age band and FAS III category (mixed models), ANCOVA-style predictor models, and correlations between module quiz performance and outcome change; subgroup and regression analyses were listed in the approved analysis plan, but the specific models were chosen at the analysis stage and the study was not registered. Location in manuscript: Methods (2.6); Results (3.2, 3.5–3.7).

Section / topic	Item	Checklist item (official descriptor)	AHOPE study – as reported
**Adverse events**	19	Summary of all important adverse events or unintended effects	No adverse events were reported during the study. Location in manuscript: Results (3.1).
**Discussion – Interpretation**	20	Interpretation accounting for bias, imprecision, multiplicity, mechanisms, implementation	The Discussion opens with the alternative explanations the design cannot exclude (repeated testing, socially desirable responding and demand characteristics, overlap between module content and AFHC items, regression to the mean) and also addresses the immediate post-test (reported intention vs enacted behaviour), the forced-choice AFHC format, curriculum–instrument divergence on low-fat items, the absence of minimally important differences, the unrecorded pre–post interval and multiplicity control. Results are framed as immediate within-participant changes in self-reported measures, without a claim of effectiveness. Location in manuscript: Discussion (4, 4.1, 4.2, 4.5); Conclusions.
**Generalisability**	21	Generalisability (external validity): population, intervention, follow-up, settings	74 schools in 19 cities limit single-school influence; fewer than half of those who began completed the session (46.7%), completers were more affluent than non-completers, and recruitment favoured digitally connected families; findings positioned as applying to Romanian adolescents and, cautiously, to the Eastern European region; no follow-up beyond the session. Location in manuscript: Discussion (4.3, 4.5); Conclusions.
**Overall evidence**	22	General interpretation of the results in the context of current evidence and theory	Results situated within the gamification and serious-games meta-analytic evidence, umbrella evidence on digital interventions, self-determination theory, and the intermediate-outcomes framing of European prevention programmes. Location in manuscript: Discussion (4.1, 4.4); Conclusions.

Note: Items 8 and 15 presuppose more than one study condition and do not apply to this single-group design; item 9 is not feasible for an open self-report intervention. Reference: Des Jarlais DC, Lyles C, Crepaz N; TREND Group. Improving the reporting quality of nonrandomized evaluations of behavioral and public health interventions: the TREND statement. Am J Public Health. 2004;94(3):361–366. doi:10.2105/AJPH.94.3.361.

Detailed Description of the AHOPE Session Content (Tables S2–S5)

Session Structure

A single AHOPE session consisted of four fixed modules ("lessons"), delivered in sequence within the digital platform and completed on the participant's own smartphone. Each module was written in Romanian in an informal, second-person register aimed at adolescents, and opened with a short hook that framed the topic in terms of everyday consequences (school concentration, sport, mood, independence). Each module was designed to take 7 to 8 minutes to read and was divided into three sections, with a progress indicator displayed at fixed points (10%, 25%, 40%, 55%, 75%, 90%, 100%). Every module ended with a 10-item multiple-choice quiz. The post-intervention questionnaire became available only once all four quizzes had been recorded as completed. Table S2 summarises the content of the four modules.

Table S2. Structure and topic coverage of the four AHOPE modules.

Module (title shown to pupils)	Section 1	Sections 2 and 3	Quiz
1. Macro- and Micronutrients: "How to turn your body into a performance machine"	Macronutrients: carbohydrates (simple vs. complex, fibre), proteins (animal and plant sources), fats (unsaturated, saturated, trans)	Micronutrients: vitamins A, C, D; iron, calcium, magnesium (function, sources, deficiency signs). Why it matters: school performance, sport, appearance, mood, independence; the 80/20 rule	10 items
2. Water, Oxygen and Sleep: "The three free things that can change your life"	Water: functions, effects of 2% dehydration, daily intake (boys 2 to 3 L, girls 1.5 to 2.5 L), practical tips, drinks that do not hydrate	Oxygen: five steps from inhalation to cellular energy; breathing technique for concentration. Sleep: the four sleep phases, effects of sleep loss, circadian rhythm, evening routine	10 items
3. Ultra-Processed Foods and Labels: "How not to be fooled by the food industry"	Real food vs. food product; the four NOVA-style processing levels; how an apple becomes an "apple-flavoured" bar	Why ultra-processed foods are problematic: empty calories, additives (colourants, preservatives, sweeteners), engineered palatability, health impact. Label masterclass: front-of-pack claims, ingredient-list rules, nutrition-facts table (portion size, sugar, fat, salt)	10 items
4. Kitchen Basics: "Why knowing how to cook is a superpower"	Five essential techniques: knife safety and cutting, boiling pasta, pan-frying, roasting vegetables, basic sauces and dressings; essential vs. optional tools; kitchen safety and hygiene rules	Smart shopping: shopping list, never shop hungry, budget, perimeter-first strategy, price per kg, cheap quality protein. Planning: batch cooking, weekly meal prep in 45 minutes	10 items

The modules used a consistent explanatory device, in which the body was compared to a smartphone (macronutrients as "chargers", water as the "operating system", micronutrients as "apps"), and each concept was followed by a concrete scenario from school or sport life (for example, a chocolate bar before a test versus oats at breakfast). Modules did not prescribe diets or calorie targets and explicitly stated that no food had to be given up; the recurring message was the "80/20 rule" (eat well most of the time, relax the rest).

End-of-Module Quizzes

Each quiz comprised 10 multiple-choice items (four options, one correct answer), presented one at a time with a visible counter ("Question 1/10"). After each response the platform displayed whether the answer was correct, the correct option, and a two- to four-

sentence explanation that referred back to the module content and stated why the distractors were wrong. Most items were framed as short situational scenarios (an exam in two hours, a football match, a supermarket comparison, raw chicken on the hands) instead of definitional recall. Quizzes could be retaken; the most recent score was retained for badge purposes. The quiz items were written specifically for the platform. No quiz item reproduced the wording of an S-NutLit, EDB or AFHC item. The curriculum and the outcome measures nevertheless address overlapping content domains (ultra-processed foods, sugar-sweetened drinks, fried food, fruit and vegetables), so part of the post-session change in those domains may reflect exposure to the taught material. The 40 question stems are listed in Table S3 in English translation; the full Romanian items with all four options and explanations are available from the corresponding author.

Mean scores on the four end-of-module quizzes were 7.4 (SD 2.4), 6.5 (SD 2.0), 8.0 (SD 1.9) and 7.6 (SD 2.0) of 10 for Macro- and Micronutrients, Water/Oxygen/Sleep, Ultra-Processed Foods and Labels, and Kitchen Basics respectively (n = 153 to 155, final attempt). Item difficulty, the proportion of participants answering correctly on their final attempt, ranged from 25.3% to 90.9% across the 40 items; the item with the lowest proportion correct in each module was item 2 in Module 1 (the muscle-repair nutrient item, 45.8%), item 1 in Module 2 (the proportion of body weight that is water, 27.1%), item 3 in Module 3 (the trans-fat labelling item, 37.0%), and item 3 in Module 4 (the pasta cooking-water item, 25.3%). Quizzes could be retaken and the most recent score was retained for badge purposes; first-attempt scores are available in the platform export and differed from the final score for 21, 29, 11 and 18 participants in Modules 1 to 4 respectively, but first-attempt performance was not used in the primary analysis.

Table S3. Question stems of the four end-of-module quizzes (translated from Romanian) with the correct option.

Module	#	Question stem	Correct option
Module 1: Macro- and Micronutrients	1	You have an important 90-minute exam in two hours. Which meal gives the most stable energy for the whole exam?	B – Oats with fruit and nuts
	2	You have just finished an intense workout and your muscles ache. Which nutrient is essential for muscle repair?	B – Proteins
	3	Which of the following is NOT a good protein source for a vegetarian?	C – French fries
	4	Trans fats, common in fast food and industrial pastry, should be:	C – Avoided as much as possible
	5	Which vitamin improves vision, especially at night, and supports skin health?	A – Vitamin A
	6	You have a cold. A friend tells you to take vitamin C to get better faster. What does the evidence actually show?	B – Starting vitamin C after symptoms begin has not been shown to work reliably; regular intake may slightly shorten a cold
	7	Dietary fibre is important because:	C – It aids digestion and prolongs satiety
	8	For strong bones and teeth, which mineral matters most during adolescence?	C – Calcium
	9	You have a football match in three hours. Which nutrient combination gives the best performance?	A – Wholegrain pasta with chicken breast

Module	#	Question stem	Correct option
	10	What is the most useful everyday approach to eating well?	C – Eat a variety of foods most of the time, and do not treat any single food as forbidden
Module 2: Water, Oxygen and Sleep	1	What percentage of your body weight is water?	B – About 60%
	2	Losing about 2% of your body water, roughly 1 litre for a 50 kg teenager, has what immediate effect?	B – Reduced concentration
	3	How much water should you drink daily as an adolescent?	C – 1.5 to 3 L, depending on sex and activity
	4	Which of the following drinks does NOT contribute efficiently to hydration?	C – Caffeinated energy drinks
	5	How long can you survive without oxygen?	B – A few minutes
	6	During a hard test you feel your brain "stops working". Which simple breathing technique can help immediately?	C – Deep breathing: inhale 5 s, hold 3 s, exhale 7 s
	7	How much sleep do you need per night?	C – 9 to 12 hours if you are under 13, and 8 to 10 hours if you are 13 or older
	8	In which sleep phase do physical repair and cell growth take place?	B – Deep sleep
	9	What is the most important rule for good-quality sleep?	B – A regular schedule
	10	What should you do in the hour before bedtime for good-quality sleep?	B – Avoid screens
Module 3: Ultra-Processed Foods and Labels	1	A cereal box says "100% NATURAL" and "SOURCE OF VITAMINS" on the front. What should you do?	B – Turn it over and read the ingredient list
	2	An ingredient list reads: "Wholemeal wheat flour, water, yeast, salt". This product is:	B – Processed, not ultra-processed
	3	A cereal label says 12 g sugar per 30 g serving. Your bowl holds 90 g. How much sugar are you eating, and where do you check?	B – 36 g, three times the declared serving; always compare the serving size on the label with the amount you actually eat
	4	Comparing two peanut butters (A: roasted peanuts, salt; B: peanuts, sugar, hydrogenated oil, molasses, salt, emulsifiers). Which is better?	A – Product A, two recognisable ingredients
	5	A "diet" drink has 0 g sugar but contains aspartame and sucralose. What does this mean?	B – Ultra-processed with artificial sweeteners; not necessarily healthier
	6	"Made with whole grains"; the ingredient list starts "Sugar, wheat flour, wholemeal wheat flour (5%)". What does this mean?	B – A marketing trick; the main ingredient is sugar
	7	A product has 35 ingredients, 20 of which you cannot pronounce. What type of food is it?	C – Ultra-processed
	8	A 30 g cereal serving has 12 g sugar; you eat a 100 g bowl. How much sugar did you consume?	C – 40 g

Module	#	Question stem	Correct option
	9	A product lists sugar as "corn syrup, brown sugar, agave nectar, molasses, sucrose". Why so many types?	C – To push sugar down the ingredient list
	10	The most important rule when buying food is:	C – Ignore the front, read the back
Module 4: Kitchen Basics	1	When cutting vegetables, how should the fingers of the hand holding the food be positioned?	B – Curled like a claw
	2	You have added oil to the pan to fry chicken. How do you know the pan is hot enough?	C – A drop of water sizzles and dances
	3	Cooking pasta for two (250 g). How much water should you use?	B – Plenty: 3 to 4 L
	4	You have just touched raw chicken. What is the mandatory next step before continuing?	C – Wash hands with soap
	5	You are taking something out of a 200 °C oven. What do you use to avoid burns?	C – Oven gloves
	6	You go shopping hungry at 18:00 after a long school day. What will most likely happen?	B – Impulse purchases of junk food
	7	You have a tight budget and want cheap, good-quality protein. Best value option?	B – Eggs / legumes
	8	Canned kidney beans: Brand A 5 lei for 500 g (10 lei/kg) vs. Brand B 7 lei for 800 g (8.75 lei/kg). Which is cheaper?	B – Brand B, cheaper per kg
	9	Sunday evening, 45 minutes for next week's meal prep. Which strategy is most efficient?	B – Partial batch cooking
	10	The most important lesson about cooking for a beginner is:	C – Master 5 to 10 simple dishes and the basic techniques

Badge and Scoring System

Each module quiz awarded a bronze, silver or gold badge according to the score (Table S4). Each badge had a module-specific name, colour and icon and displayed a one-line motivational message. Module scores were summed to a total out of 40, which determined one of three final badges (Table S5). The badges were the only "competitive and collaborative feature" of the platform; no peer leaderboards, team tasks or social comparison were implemented, and no participant could see another participant's score.

Table S4. Module-level badges and score thresholds.

Level	Score on 10-item quiz	Module 1	Module 2	Module 3	Module 4
Bronze	6 to 7 of 10	Nutrition Explorer	Basics Learner	Label Reader	Kitchen Starter
Silver	8 of 10	Fuel Specialist	Vitality Keeper	Smart Consumer	Cooking Warrior
Gold	9 to 10 of 10	Energy Master	Foundation Expert	Ingredient Detective	Kitchen Rebel

Table S5. Final (session-level) badges.

Final badge	Total score (max 40)	Condition
Nutrition Apprentice (bronze)	24 to 31 (60 to 77%)	At least 6 of 10 on every module
Nutrition Specialist (silver)	32 to 35 (80 to 87%)	Average 8 to 8.75 per module
Nutrition Champion (gold)	36 to 40 (90 to 100%)	Average 9 to 10 per module

Note: Final badges were assigned by total score; the module-level condition shown in the table was advisory and displayed to the pupil to summarise performance on each module, but did not override the total-score badge. In the analytic sample (n = 153, participants with all four module scores available), total scores ranged from 8 to 40 of 40; 26 participants (17.0%) scored below 24, the nominal threshold for the lowest badge band, and 42 participants (27.5%) whose total score qualified for a badge did not meet the module-level condition of at least 6 of 10 on every module. The final badge shown to the pupil was therefore determined by total score alone.

No teacher-led instruction, worksheet or other non-digital material accompanied any module. Supervising teachers were instructed that their role was limited to ensuring device access and on-task behaviour, and that they should not present or explain module content. Adherence to this instruction was not independently observed, and sessions completed outside school during the summer holiday were unsupervised.

Table S6. *Item-level change on the Adolescent Food Habits Checklist (AFHC), ranked by significance.*

Item	% healthy, Pre	% healthy, Post	p_BH
2 – Usually avoids fried foods	34.8	62.6	<0.001
7 – Avoids sausages/burgers	45.8	68.4	<0.001
13 – Tries to eat plenty of fruit and vegetables	57.4	76.1	<0.001
3 – Eats a dessert/pudding if available (reverse-scored)	32.3	51.0	0.001
11 – If having dessert at home, chooses something low in fat	46.5	65.2	0.001
4 – Eats at least one serving of fruit daily	61.3	78.1	0.002
16 – Chooses a diet soft drink	49.0	65.8	0.002
9 – Tries to keep overall sugar intake down	49.0	65.8	0.004
6 – Chooses a low-fat crisps brand	43.9	60.0	0.005
20 – Chooses the healthiest dessert in a restaurant	49.7	65.2	0.005
22 – Eats at least three servings of fruit most days	45.2	60.6	0.005
10 – Eats at least one serving of vegetables/salad daily	61.9	75.5	0.005
15 – Eats vegetables/salad with the evening meal	62.6	76.1	0.012
12 – Rarely eats takeaway meals	51.6	65.8	0.013
21 – Often has cream on desserts (reverse-scored)	60.0	72.3	0.023
5 – Tries to keep overall fat intake down	52.9	63.9	0.036
8 – Often buys pastries or cakes (reverse-scored)	45.2	56.1	0.046
23 – Generally tries to have a healthy diet	74.2	81.3	0.140
18 – Packed lunch usually includes chocolate/biscuits (reverse-scored)	44.5	52.9	0.143
1 – Chooses a low-fat option for lunch away from home	38.1	43.9	0.369
19 – Chooses fruit for between-meal snacks	60.6	64.5	0.544
14 – Often eats sweet snacks between meals (reverse-scored)	41.3	44.5	0.636
17 – Spreads butter/margarine thinly on bread	61.9	63.2	0.883

Note: AFHC administered in an adapted forced-choice format without the original "not applicable" option. p_BH from McNemar's test for paired binary data, Benjamini-Hochberg corrected across the 23 items. Item wording follows the original Adolescent Food Habits Checklist (Johnson et al., 2002); items 3, 8, 14, 18, and 21 are reverse-worded (a 'False' response counts as the healthy answer) and were reverse-scored before all other analyses in this paper.

Table S7. *Sex differences in the size of the pre-to-post change, and time × moderator interaction tests.*

Outcome	Δ Boys	Δ Girls	p (boys vs. girls)	Sex × time p_BH	Age × time p_BH	FAS × time p_BH
S-NutLit	0.79	0.78	0.970	0.970	0.901	0.901
EDB total	3.84	4.84	0.464	0.901	0.901	0.901
EDB personal	1.37	1.56	0.702	0.970	0.970	0.901
EDB social	1.56	1.58	0.970	0.970	0.970	0.901
EDB global	0.91	1.69	0.132	0.901	0.901	0.971
AFHC total	3.41	2.82	0.511	0.901	0.901	0.901

Note: Δ, mean within-person change (post minus pre) in each sex, from the linear mixed-effects model; p (boys vs. girls), test of the difference in change between boys and girls; sex × time, age × time and FAS × time p_BH, interaction p-values after Benjamini-Hochberg correction across the six outcomes. Age bands 10 to 14 vs. 15 to 19 years; FAS III categories low, medium and high. Simple contrasts within each subgroup are given in Table S8.

Table S8. Simple contrasts of the pre-to-post change within levels of sex, age band and family affluence (linear mixed-effects models).

Outcome	Subgroup	n	Δ	95% CI	p
S-NutLit	Boys	70	0.79	[0.54, 1.04]	<0.001
S-NutLit	Girls	85	0.78	[0.55, 1.01]	<0.001
EDB total	Boys	70	3.84	[1.86, 5.82]	<0.001
EDB total	Girls	85	4.84	[3.04, 6.63]	<0.001
EDB personal	Boys	70	1.37	[0.63, 2.11]	<0.001
EDB personal	Girls	85	1.56	[0.90, 2.23]	<0.001
EDB social	Boys	70	1.56	[0.80, 2.32]	<0.001
EDB social	Girls	85	1.58	[0.89, 2.27]	<0.001
EDB global	Boys	70	0.91	[0.16, 1.67]	0.018
EDB global	Girls	85	1.69	[1.01, 2.38]	<0.001
AFHC total	Boys	70	3.41	[2.10, 4.73]	<0.001
AFHC total	Girls	85	2.82	[1.63, 4.02]	<0.001
S-NutLit	10 to 14 years	56	0.68	[0.39, 0.96]	<0.001
S-NutLit	15 to 19 years	99	0.85	[0.63, 1.06]	<0.001
EDB total	10 to 14 years	56	4.86	[2.64, 7.07]	<0.001
EDB total	15 to 19 years	99	4.12	[2.46, 5.79]	<0.001
EDB personal	10 to 14 years	56	1.43	[0.60, 2.25]	<0.001
EDB personal	15 to 19 years	99	1.51	[0.88, 2.13]	<0.001
EDB social	10 to 14 years	56	1.62	[0.78, 2.47]	<0.001
EDB social	15 to 19 years	99	1.54	[0.90, 2.17]	<0.001
EDB global	10 to 14 years	56	1.80	[0.96, 2.65]	<0.001
EDB global	15 to 19 years	99	1.08	[0.45, 1.71]	<0.001
AFHC total	10 to 14 years	56	3.70	[2.23, 5.16]	<0.001
AFHC total	15 to 19 years	99	2.75	[1.65, 3.85]	<0.001
S-NutLit	Low affluence	18	0.55	[0.05, 1.04]	0.032
S-NutLit	Medium affluence	42	0.79	[0.46, 1.11]	<0.001
S-NutLit	High affluence	95	0.83	[0.61, 1.05]	<0.001
EDB total	Low affluence	18	2.28	[-1.63, 6.18]	0.251
EDB total	Medium affluence	42	5.02	[2.47, 7.58]	<0.001

Outcome	Subgroup	n	Δ	95% CI	p
EDB total	High affluence	95	4.51	[2.81, 6.21]	<0.001
EDB personal	Low affluence	18	0.56	[-0.90, 2.01]	0.451
EDB personal	Medium affluence	42	1.67	[0.72, 2.62]	<0.001
EDB personal	High affluence	95	1.57	[0.94, 2.20]	<0.001
EDB social	Low affluence	18	0.78	[-0.72, 2.27]	0.306
EDB social	Medium affluence	42	1.86	[0.88, 2.84]	<0.001
EDB social	High affluence	95	1.59	[0.94, 2.24]	<0.001
EDB global	Low affluence	18	0.94	[-0.55, 2.44]	0.215
EDB global	Medium affluence	42	1.50	[0.52, 2.48]	0.003
EDB global	High affluence	95	1.35	[0.70, 2.00]	<0.001
AFHC total	Low affluence	18	4.56	[1.99, 7.12]	<0.001
AFHC total	Medium affluence	42	4.00	[2.32, 5.68]	<0.001
AFHC total	High affluence	95	2.41	[1.29, 3.53]	<0.001

Note: Δ, estimated marginal mean of the within-person change (post minus pre) in the subgroup; 95% CI, confidence interval with Satterthwaite-approximated degrees of freedom; p, test of the change within the subgroup. Interaction tests are given in Table S7.

Table S9. ANCOVA-adjusted predictors of post-intervention scores (baseline score, sex, age, FAS III score, physical-activity days and prior nutrition-education exposure).

Outcome	Baseline score, β (p)	Sex (F vs. M), β (p)	Age, β (p)	FAS score, β (p)	PA days, β (p)	Prior exposure, β (p)	R^2 (adjusted R^2)
S-NutLit	0.33 (<0.001)	−0.03 (0.814)	−0.04 (0.160)	0.081 (0.001)	0.093 (0.026)	−0.070 (0.487)	28.5% (25.1%)
EDB total	0.32 (<0.001)	1.21 (0.258)	−0.57 (0.016)	0.47 (0.025)	0.52 (0.126)	1.045 (0.214)	24.6% (21.0%)
EDB personal	0.23 (0.001)	0.37 (0.344)	−0.15 (0.075)	0.23 (0.002)	0.23 (0.065)	0.439 (0.144)	21.7% (18.0%)
EDB social	0.25 (<0.001)	0.20 (0.614)	−0.12 (0.154)	0.17 (0.031)	0.15 (0.223)	0.408 (0.177)	16.8% (12.9%)
EDB global	0.44 (<0.001)	0.68 (0.117)	−0.31 (0.002)	0.08 (0.312)	0.16 (0.235)	0.177 (0.607)	29.2% (25.8%)
AFHC total	0.34 (<0.001)	−0.61 (0.409)	−0.27 (0.099)	0.04 (0.788)	0.46 (0.060)	−0.587 (0.290)	21.3% (17.5%)

Note: β, unstandardised regression coefficient for the corresponding predictor in the model for that outcome. PA, physical activity; FAS, Family Affluence Scale III. R^2, proportion of variance in the post-intervention score explained by the full model, with adjusted R^2 in parentheses. Prior nutrition-education exposure was entered as a three-level factor; the tabulated coefficient is the linear-trend estimate (0 = none, 1 = very little, 2 = several activities) from an otherwise identical model.

Table S10. Baseline characteristics of participants who completed the session and those who did not.

Variable	Completers (n = 155)	Non-completers (n = 177)	p
Age, years	15.3 (2.3)	15.5 (1.8)	0.32
Girls, %	54.8	51.4	0.53
FAS III score (0–13)	10.0 (2.7)	8.9 (2.6) a	<0.001
S-NutLit, baseline (1–5)	3.05 (1.06)	3.26 (0.73) a	0.034
EDB total, baseline (6–36)	20.99 (8.18)	22.62 (6.75) a	0.050
AFHC total, baseline (0–23)	11.70 (5.23)	11.48 (5.06) a	0.71

Note: Values are mean (SD) unless stated otherwise; p from Welch's t-test (continuous variables) or the chi-squared test (sex). FAS III, Family Affluence Scale III; S-NutLit, Short Nutrition Literacy Scale; EDB, Engagement in Dietary Behaviour; AFHC, Adolescent Food Habits Checklist (adapted forced-choice version). a n = 176, because one non-completer record had a data-entry misalignment.
